# Prospective Validation of American Diabetes Association Risk Tool for Predicting Pre-Diabetes and Diabetes in Taiwan–Taichung Community Health Study

**DOI:** 10.1371/journal.pone.0025906

**Published:** 2011-10-05

**Authors:** Chia-Ing Li, Ling Chien, Chiu-Shong Liu, Wen-Yuan Lin, Ming-May Lai, Cheng-Chun Lee, Fei-Na Chen, Tsai-Chung Li, Cheng-Chieh Lin

**Affiliations:** 1 Department of Medical Research, China Medical University Hospital, Taichung, Taiwan; 2 School of Medicine, College of Medicine, China Medical University, Taichung, Taiwan; 3 Institute of Public Health, College of Public Health, China Medical University, Taichung, Taiwan; 4 Graduate Institute of Biostatistics, College of Public Health, China Medical University, Taichung, Taiwan; 5 Department of Family Medicine, China Medical University Hospital, Taichung, Taiwan; 6 Department of Social Medicine, College of Medicine, China Medical University, Taichung, Taiwan; 7 Department of Family Medicine, College of Medicine, China Medical University, Taichung, Taiwan; 8 Department of Neurology, China Medical University Hospital, Taichung, Taiwan; 9 Graduate Institute of China Medical Science, College of Chinese Medicine, China Medical University, Taichung, Taiwan; 10 Biostatistics Center, China Medical University, Taichung, Taiwan; 11 Institute of Health Care Administration, College of Health Science, Asia University, Taichung, Taiwan; 12 School and Graduate Institute of Health Care Administration, College of Public Health, China Medical University, Taichung, Taiwan; Universidad Peruana Cayetano Heredia, Peru

## Abstract

**Background:**

A simple diabetes risk tool that does not require laboratory tests would be beneficial in screening individuals at higher risk. Few studies have evaluated the ability of these tools to identify new cases of pre-diabetes. This study aimed to assess the ability of the American Diabetes Association Risk Tool (ADART) to predict the 3-year incidence of pre-diabetes and diabetes in Taiwanese.

**Methods:**

This was a 3-year prospective study of 1021 residents with normoglycemia at baseline, gathered from a random sample of residents aged 40–88 years in a metropolitan city in Taiwan. The areas under the curve (AUCs) of three models were compared: ADART only, ADART plus lifestyle behaviors at baseline, and ADART plus lifestyle behaviors and biomarkers at baseline. The performance of ADART was compared with that of 16 tools that had been reported in the literature.

**Results:**

The AUCs and their 95% confidence intervals (CIs) were 0.60 (0.54–0.66) for men and 0.72 (0.66–0.77) for women in model 1; 0.62 (0.56–0.68) for men and 0.74 (0.68–0.80) for women in model 2; and 0.64 (0.58–0.71) for men and 0.75 (0.69–0.80) for women in model 3. The AUCs of these three models were all above 0.7 in women, but not in men. No significant difference in either women or men (p = 0.268 and 0.156, respectively) was observed in the AUC of these three models. Compared to 16 tools published in the literature, ADART had the second largest AUC in both men and women.

**Conclusions:**

ADART is a good screening tool for predicting the three-year incidence of pre-diabetes and diabetes in females of a Taiwanese population. The performance of ADART in men was similar to the results with other tools published in the literature. Its performance was one of the best among the tools reported in the literature.

## Introduction

Diabetes is an important public health problem in the world [1], even in developing countries [2]. In Lin's study, using WHO diagnostic criteria, the prevalence rates of diabetes and impaired glucose regulation (IGR) were 9.51% (male, 10.08%; female, 9.14%) and 14.40% (male, 14.48%; female, 14.35%) respectively in Fujian province, southeast China [2]. The prevalence of type 2 diabetes in middle-aged adults in Taiwan increased steadily from 5.1% to 8.2% to 12.8% in 1970, 1986 and 1993, respectively [3], [4]. Among men aged 65 years and above, as reflected in the National Nutrition Survey in Taiwan, the prevalence increased dramatically from 13.1% to 17.6% to 28.5% in 1993–1996, 2002 and 2005–2008, respectively [4]. Newly diagnosed diabetes was found in 53.44% of diabetes subjects [3].

Diabetes has become one of the most challenging diseases threatening the public [5], hence early screening and effective prevention of diabetes has become a major public health issue. If we can prevent diabetes in the early stage, then we can take action against the disease and disability, and reduce complications and even death. To increase the sensitivity of the diagnostic test, the American Diabetes Association (ADA) lowered the cutoff for IFG from 110 to 100 mg/dl [6]; it was estimated that the number of Americans thought to have “pre-diabetes” was 41 million, using this cutoff point [7].

A simple diabetes risk tool that does not require any laboratory tests would be beneficial in screening individuals at higher risk. Previous cross-sectional or longitudinal screening studies have evaluated the performance of questionnaire-based screening tools in identifying the prevalence or incidence of diabetes (8–23); however, few studies have evaluated the ability of those tools to identify new cases of pre-diabetes.

The ADA has proposed a risk tool for screening diabetes [24], but its performance for screening pre-diabetes has never been reported. To remedy this, we have set three aims for this study. First, we aimed to evaluate the performance of the American Diabetes Association Risk Tool (ADART) in identifying 3-year incident cases of pre-diabetes and diabetes in a prospective cohort study of Taiwanese aged 40–88 years in a metropolitan city in Taiwan. Second, we compared its performance with that of ADART plus lifestyle behaviors at baseline, and ADART plus lifestyle behaviors and biomarkers at baseline in this sample. Third, we compared the performance of ADART in identifying the incidence of pre-diabetes and diabetes with that of 16 diabetes screening tools that had been reported in the literature.

## Methods

### Study population

This was a longitudinal epidemiological study based on data from the Taichung Community Health Study (TCHS). At baseline, a total of 2359 residents of Taichung City in Taiwan, aged 40 and over, were randomly selected in October 2004 using multistage sampling [25]. During the period April 2007 to June 2009, the original participants were invited to take part in a follow-up examination, and 1631 of the 2359 original participants agreed to participate. Among them, 610 (37%) were excluded from the analysis because they either had a history of diabetes mellitus or had evidence of pre-diabetes (FPG ≥100 mg/dl, according to the ADA). Therefore, the study population comprised 1021 individuals with normal blood glucose levels. This study was approved by the Human Research Committee of the China Medical University Hospital and written informed consent was obtained from each participant.

### Data collection

Anthropometric measurements were obtained during the complete physical examination. Weight and height were measured on an auto-anthropometer (super-view, HW-666) while the subjects were shoeless and wearing light clothing. Body mass index (BMI) was defined as weight in kilograms divided by height in meters squared. With the participant standing, waist circumference was measured midway between the superior iliac crest and the costal margin.

Blood pressure was measured using an electronic device (COLIN, VP-1000, Japan) three times after the subjects had rested for 20 minutes. The lowest systolic and diastolic blood pressure was recorded. Blood was drawn from an antecubital vein in the morning after a 12-hour overnight fast and was sent for analysis within four hours of blood collection. Biochemical markers such as fasting plasma glucose, high-density lipoprotein cholesterol (HDL-C), triglyceride, urine albumin, and creatinine were analyzed with a biochemical autoanalyzer (Beckman Coluter Synchron System, Lx-20, Fullerton, CA, USA) at the Clinical Laboratory Department of China Medical University Hospital. The interassay and intraassay CVs for fasting plasma glucose were 4% and 4%, respectively. We measured cholesterol and triglyceride in serum mode. Triglyceride levels were determined by an enzymatic colorimetric method. The HDL-C level was measured using a direct HDL-C method, and the low-density lipoprotein cholesterol (LDL-C) level was measured using a direct LDL-C method.

Data on sociodemographic characteristics, including gender, smoking, drinking, betel nut chewing, physical activity, time spent watching TV every week, family history of diabetes, family history of cardiovascular-related diseases, physician-diagnosed diseases, and medication history were collected during the complete physical examination. Information regarding time spent watching TV was obtained using the open question “On average, how many hours a day (or a week) do you spend watching TV?”

### American Diabetes Association Risk Tool

The ADART was constructed according to the 2004 criteria for screening pre-diabetes [24]. The screening tool comprises eight self-reported items for both men and women, including age ≥45 years, BMI ≥25 kg/m^2^, family history of diabetes, race or ethnicity, level of physical activity, previously identified IFG or IGT, high blood pressure, HDL cholesterol <$>\raster="rg3"<$>≦35 mg/dl (0.90 mmol/l) and/or triglyceride level ≥250 mg/dl (2.82 mmol/l), and history of vascular disease. There are two additional items for women: history of gestational diabetes mellitus (GDM) or delivery of a baby weighing >4000 grams (9 lbs), and the presence of polycystic ovary syndrome. In this study, we did not take race or ethnicity into account.

### Statistical analysis

Baseline characteristics of individuals who were followed up and those who were not were compared using standardized mean differences, calculated as the difference in means of a variable divided by a pooled estimate of the standard deviation of the variable. This measure is not influenced by sample size and is useful for comparing cohorts in large observational studies. A value of 0.1 SD or less indicates a negligible difference in means between groups [26]. Differences in proportions were assessed using the Chi-square test. To validate the performance of ADART combined with different diabetes risk factors, we derived three logistic regression models: ADART only, ADART plus lifestyle behaviors at baseline, and ADART plus lifestyle behaviors and biomarkers at baseline. Those variables which were statistically significant at a level of 0.25 were brought into the models [27]. A nonparametric method was used to test whether the areas under the curve (AUCs) for each receiver operating characteristic curve of these three models or among different tools were different [28]. Determination of the optimal cutoff points that could be used to detect pre-diabetes or diabetes was based on the Youden index. We also calculated the net reclassification improvement (NRI) and integrated discrimination improvement (IDI) of the models that included ADART plus lifestyle behaviors at baseline or ADART plus lifestyle behaviors and biomarkers at baseline compared with the model with ADART only according to the method of Pencina et al. [29]. For NRI, four risk categories were chosen a priori: very low risk (<10%), low risk (10–20%), intermediate (20–30%) and high risk (>30%).

## Results

In general, there were no significant differences in distributions of sociodemographic variables, anthropometric measurements, or levels of biomarkers between the men and women who were followed up and those who were not (Table 1). Of the 1021 participants in this sample, 184 (18%) had elevated FPG levels (≧100 mg/dl) during the three-year follow-up period. Men with abnormal FPG levels had lower monthly incomes, but had a higher prevalence of family history of hyperlipidemia, higher diastolic blood pressure and triglyceride levels than men with normal levels of FPG. Women with abnormal FPG levels had lower levels of education but higher weight, larger waist size, higher BMI, systolic and diastolic blood pressure, triglyceride levels and higher Framingham scores than women with normal FPG levels.

**Table 1:** Baseline characteristics in individuals who were followed up and those who were not according to gender.

	Men (n = 1116)			Women (n = 1195)		
	Not followedn = 286mean (SD)	Followedn = 830mean (SD)	standardized mean differences	Not followedn = 394mean (SD)	Followedn = 801mean (SD)	standardized mean differences
Age (year)	59.29(13.26)	57.78(11.66)	0.007	55.96(11.63)	54.45(9.51)	−0.012
Weight (kg)	67.62(10.51)	69.29(10.18)	−0.009	58.60(8.97)	57.25(8.36)	−0.009
Height (cm)	166.13(6.13)	166.63(6.15)	−0.005	154.90(5.39)	155.62(5.29)	−0.002
FAT (%)	25.86(5.84)	26.01(5.58)	−0.002	37.02(6.19)	35.83(5.88)	−0.010
SYS (mmHg)	141.04(21.10)	137.83(20.09)	0.009	136.29(24.63)	130.37(21.15)	−0.011
DIA (mmHg)	83.37(12.33)	82.22(11.03)	0.006	77.22(12.85)	74.51(11.93)	−0.010
Waist (cm)	85.87(9.01)	86.47(8.65)	−0.004	78.14(9.50)	75.97(8.39)	−0.007
GOT (IU/L)	28.78(26.04)	27.47(11.88)	0.003	25.93(15.67)	25.64(14.84)	−0.036
GPT (IU/L)	30.95(48.61)	29.91(19.26)	0.001	25.56(30.62)	24.62(22.06)	−0.071
CHOL (mg/dl)	197.88(39.06)	201.67(35.99)	−0.006	205.94(40.27)	206.11(37.48)	−0.012
TG (mg/dl)	128.63(97.06)	140.16(117.34)	−0.007	112.92(76.73)	104.37(66.66)	−0.040
FPG (mg/dl)	110.46(41.05)	104.83(24.74)	0.008	104.91(35.60)	98.96(21.65)	−0.020
WBC (10^3^/µl)	6.53(1.88)	7.55(38.75)	−0.022	5.80(1.64)	5.59(1.47)	−0.017
RBC (10^6^/µl)	4.94(0.57)	5.00(0.54)	−0.006	4.51(0.45)	4.54(0.46)	−0.006
HGB (g/dl)	14.82(1.32)	15.05(1.18)	−0.010	13.25(1.25)	13.21(1.22)	−0.006
HCT (%)	44.27(3.70)	44.86(3.29)	−0.009	40.27(3.27)	40.20(3.21)	−0.005
PLT (10^3^/µl)	227.90(59.52)	224.29(57.10)	0.004	244.73(63.17)	247.67(57.99)	−0.015
URIC (mg/dl)	6.37(1.42)	6.30(1.39)	0.003	5.24(1.22)	4.94(1.06)	−0.014
HDL (mg/dl)	41.50(10.84)	41.28(10.61)	0.001	49.04(12.36)	50.80(12.78)	−0.015
LDL (mg/dl)	126.56(37.00)	128.22(32.77)	−0.003	128.39(34.37)	127.13(33.49)	−0.016
BUN (mg/dl)	14.50(6.29)	13.87(4.28)	0.006	12.84(4.82)	11.97(3.91)	−0.022
MA (mg/g cr)	39.58(209.28)	25.20(100.26)	0.004	28.90(77.33)	20.22(90.65)	−0.158
Creatine (mg/dl)	1.11(0.63)	1.05(0.25)	0.006	0.81(0.46)	0.73(0.17)	−0.033

SD: standard deviation.

Model 1 showed that of the eight self-reported ADART variables, only a history of cardiovascular disease was associated with an increased incidence of abnormal FPG in men (OR = 2.71, p<0.01) (Table 2). Model 1 also revealed that the likelihood of having abnormal FPG levels was higher in women with BMI<$>\raster="rg2"<$>25 kg/m^2^ (OR = 2.59, p<0.001), HDL <35 mg/dl or TG<$>\raster="rg2"<$>250 mg/dl (OR = 4.27, p<0.001), or gestational diabetes, or in women who delivered a neonate weighing >4000g (OR = 1.98, p<0.05). In model 2, we further considered family history and lifestyle behaviors. Men with a family history of hyperlipidemia were at increased risk of abnormal FPG at a level of significance of 0.25. Women who had less than 9 years of education and those who watched TV for greater than or equal to 25 hours per week were at significantly increased risk of abnormal FPG.

**Table 2:** The ability of ADART plus lifestyle behaviors and biomarkers at baseline for predicting 3-year incidence of pre-diabetes and diabetes.

	OR					
	Men (n = 456)			Women (n = 565)		
	model 1	model 2	model 3	model 1	model 2	model 3
ADART						
age≥45	1.53(0.79, 2.96)	1.57(0.81, 3.04)	1.55(0.79, 3.02)	1.48(0.69, 3.15)	1.17(0.54, 2.52)	1.15(0.53, 2.49)
BMI≥25	1.03(0.63, 1.68)	1.06(0.65, 1.73)	1.02(0.62, 1.67)	2.59(1.52, 4.43)***	2.16(1.25, 3.75)**	2.08(1.19, 3.92)**
family history of diabetes	1.10(0.63, 1.93)	1.00(0.56, 1.77)	0.98(0.55, 1.75)	1.49(0.86, 2.59)	1.60(0.90, 2.82)	1.63(0.92, 2.89)
low physical activity level	1.05(0.64, 1.72)	1.06(0.65, 1.74)	1.04(0.63, 1.71)	0.79(0.45, 1.38)	0.74(0.41, 1.30)	0.74(0.41, 1.31)
previously identified IFG or IGT	1.93(0.33, 11.21)	2.02(0.34, 11.86)	2.05(0.35, 12.18)	2.68(0.18, 40.26)	3.50(0.24, 50.26)	3.06(0.22, 42.77)
high blood pressure	1.37(0.83, 2.27)	1.28(0.77, 2.14)	1.24(0.74, 2.08)	1.17(0.64, 2.11)	1.18(0.65, 2.14)	0.97(0.50, 1.88)
HDL cholesterol≤35 or TG≥250 (mg/dl)	0.74(0.45, 1.24)	0.74(0.44, 1.25)	0.62(0.36, 1.07)	4.27(2.09, 8.75)***	4.35(2.10, 9.01)***	4.46(2.14, 9.32)***
history of cardiovascular disease	2.71(1.36, 5.37)**	2.72(1.37, 5.41)**	2.96(1.47, 5.97)**	0.81(0.28, 2.32)	0.78(0.27, 2.30)	0.79(0.27, 2.35)
history of GDM or delivery of a baby	-#	-#	-#	1.98*(1.04, 3.78)	2.04(1.06, 3.93)*	2.05*(1.06, 3.95)
weighing>4000 g						
with polycystic ovary syndrome	-#	-#	-#	1.36(0.50, 3.72)	1.54(0.56, 4.23)	1.64(0.59, 4.52)
family history of hyperlipidemia	-	1.87(0.96, 3.65)	1.74(0.89, 3.42)	-	-	-
education attainment≤9 years	-	-	-	-	1.90*(1.11, 3.25)	1.83*(1.07, 3.14)
TV watching time≥25 hrs/week	-	-	-	-	1.95*(1.13, 3.37)	1.92*(1.11, 3.33)
baseline triglyceride>150 (mg/dl)	-	-	1.96*(1.17, 3.28)	-	-	-
baseline diastolic blood pressure≥85 mmHg	-	-	-	-	-	1.65(0.83, 3.27)

*p<0.05;

**p<0.01;

***p<0.001. -#: OR were not available because the items of ADART were only for women; -: OR were not available because the variables did not reach the level of significance set for entering into models. ADART: American Diabetes Association Risk Tool.

Model 3, which took ADART plus lifestyle behaviors and biomarkers at baseline into account, revealed that a history of cardiovascular disease and hypertriglyceride at baseline were significant variables in the final model in men; in women, however, there were no additional significant variables.

**Figure 1:**
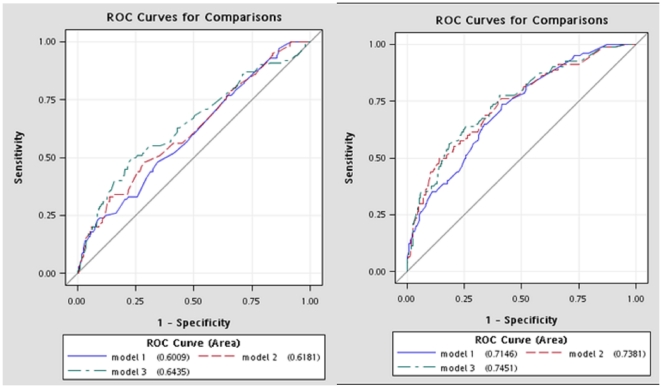
A—Comparing the AUCs of model 1, model 2, and model 3 in men. B—Comparing the AUCs of model 1, model 2, and model 3 in Women.

The areas under the receiver operating characteristic curves (AUC) for these three multivariate models were similar in men (AUC = 0.60, 95% CI = 0.54–0.66 for model 1; AUC = 0.62, 95% CI = 0.56–0.68 for model 2; and AUC = 0.64, 95% CI = 0.58–0.71 for model 3; p value for overall test: 0.268) (Figure 1A). The AUC for these three multivariate models were also similar in women (AUC = 0.72, 95% CI = 0.66–0.77 for model 1; AUC = 0.74, 95% CI = 0.68–0.80 for model 2; and AUC = 0.75, 95% CI = 0.69–0.80 for model 3; p value for overall test: 0.156) (Figure 1B); however, they were all above 0.7 and were much larger than those for men. Using the Youden index to determine the optimal cutoff points, we found that the sensitivity was 0.77 for men and 0.76 for women, and that the specificity was 0.35 for men and 0.54 for women (Table 3). In men, net reclassification improved by 1.5% when family history of hyperlipidemia was entered (model 2) and improved by 9.6% when baseline triglyceride was further entered (model 3) (p = 0.9538 and 0.7862, respectively). The integrated discrimination improved by 0.007 and 0.008 for models 2 and 3, respectively (p = 0.1414 and 0.0041, respectively). In women, net reclassification improved by 0.3% when education and time for TV watching were entered (model 2) and improved by 5.0% when baseline diastolic blood pressure was further entered (model 3) (p = 0.1037 and 0.9055, respectively). The integrated discrimination improved by 0.030 and 0.034 for models 2 and 3, respectively (p = 0.0044 and 0.0028, respectively).

**Table 3:** The predictive performance of American Diabetes Association Risk Tool.

Model	AUC (95% CI)	p value	sensitivity	specificity	LR^+^	LR^-^	Youdenindex	predicted probability^#^	NRI	p value for NRI	IDI	p value for IDI
Male												
model 1	0.60 (0.54–0.66)	-	0.77	0.35	1.19	0.65	0.12	0.2804	-	-	-	-
model 2	0.62 (0.56–0.68)	0.3171	0.78	0.34	1.19	0.64	0.12	0.3829	0.015	0.9538	0.007	0.1414
model 3	0.64 (0.58–0.71)	0.1055	0.71	0.45	1.28	0.65	0.16	0.2384	0.096	0.7862	0.008	0.0041
Female												
model 1	0.72 (0.65–0.77)	-	0.76	0.54	1.64	0.44	0.30	0.1181	-	-	-	-
model 2	0.74 (0.68–0.80)	0.2126	0.75	0.60	1.86	0.42	0.35	0.2370	0.003	0.1037	0.030	0.0044
model 3	0.75 (0.69–0.80)	0.0862	0.74	0.62	1.94	0.42	0.36	0.1626	0.050	0.9055	0.034	0.0028

model 1: ADART, model 2: ADART+lifestyle behaviors at baseline, model 3: ADART+lifestyle behaviors+biomarkers; LR^+^ = positive likelihood ratio test; LR^-^ = negative likelihood ratio test; Youden index was defined as the maximum of (sensitivity+specificity-1); #: predicted probability for the optimal cutoff points; NRI: net reclassification improvement; IDI: integrated discrimination improvement.

Data on the predictive performance of the 16 screening tools for pre-diabetes and diabetes in our study are summarized in Table 4. The largest AUC for pre-diabetes and diabetes in men was 0.64 (95% CI: 0.58–0.70), developed by Schmidt, with 56% sensitivity and 67% specificity using optimal cutoff values. The AUCs of the ROC for pre-diabetes and diabetes using the ADA tool were significantly greater than those for the tools developed by Ramachandran, Aekplakorn, Lawati, Balkau, Bindraban, but there was no statistical difference in the AUCs of the ROC between the ADA tool and the tools developed by Baan, Griffin, Stern, Lindström, Glumer, Mohan, Schulze, de León, Cox, Wilson, and Schmidt. The largest AUC of the ROC for pre-diabetes and diabetes in women was 0.72 (95% CI: 0.65–0.77), with 74% sensitivity and 58% specificity. The AUCs for the ADA tool were significantly greater than for the tools developed by Baan PM1, Lindström, Glumer, Mohan, Romachandran, Lawati, Schulze, Balkau, Bindraban, and Wilson, but there were no statistical differences in the AUCs between the ADA tool, and the tools developed by Baan PM2, Griffin, Stern, Aekplakorn, de León, Cox, and Schimidt for pre-diabetes and diabetes.

**Table 4:** ADART and instruments published in literature in screening undiagnosed pre-diabetes and diabetes.

Tools	AUC (95%CI)	sensitivity	specificity	LR+	LR-	Youden index
Men						
ADA^24^	0.60(0.54–0.66)	0.24	0.90	2.47	0.84	0.14
Baan^14^						
PM1	0.57(0.51–0.63)	0.77	0.35	1.18	0.66	0.12
PM2	0.54(0.48–0.60)	0.90	0.18	1.10	0.54	0.08
Griffina, 12	0.54(0.47–0.60)	0.69	0.38	1.11	0.82	0.07
Stern^b, 15^	0.60(0.54–0.66)	0.72	0.45	1.30	0.63	0.17
Lindström^16^	0.55(0.48–0.61)	0.86	0.23	1.12	0.61	0.09
Glümer^23^	0.56(0.50–0.62)	0.55	0.58	1.30	0.78	0.13
Mohan^8^	0.53(0.47–0.59)	0.96	0.10	1.07	0.39	0.06
Ramachandran^9^	0.51(0.44–0.57)*	0.27	0.79	1.28	0.92	0.06
Schmidt^b, 17^	0.64(0.58–0.70)	0.56	0.67	1.71	0.65	0.23
Aekplakorn^18^	0.50(0.44–0.57)*	0.27	0.77	1.19	0.94	0.04
Lawati^10^	0.52(0.46–0.58)*	0.18	0.87	1.35	0.95	0.05
Schulze^c, 19^	0.55(0.49–0.61)	0.73	0.40	1.22	0.67	0.13
León^11^	0.57(0.51–0.63)	0.74	0.44	1.32	0.59	0.18
Wilson^20^	0.54(0.48–0.60)	0.71	0.38	1.14	0.77	0.09
Balkau^22^	0.50(0.44–0.56)*	0.82	0.21	1.03	0.87	0.03
Bindraban^21^	0.53(0.47–0.59)*	0.71	0.35	1.09	0.84	0.06
Cox^13^	0.52(0.46–0.59)	0.09	0.95	1.83	0.96	0.04
Women						
ADA^24^	0.72(0.65–0.77)	0.74	0.58	1.76	0.45	0.32
Baan^14^						
PM1	0.58(0.52–0.64)*	0.35	0.76	1.47	0.86	0.11
PM2	0.69(0.64–0.75)	0.80	0.52	1.65	0.39	0.31
Griffina, 12	0.66(0.60–0.72)	0.74	0.52	1.55	0.50	0.26
Stern^b, 15^	0.73(0.67–0.79)	0.71	0.65	2.02	0.44	0.36
Lindström^16^	0.62(0.55–0.69)*	0.30	0.87	2.28	0.81	0.17
Glumer^23^	0.62(0.56–0.69)*	0.54	0.67	1.60	0.70	0.20
Mohan^8^	0.53(0.46–0.60)*	0.14	0.91	1.55	0.94	0.05
Ramachandran^9^	0.64(0.58–0.71)*	0.63	0.58	1.52	0.63	0.21
Schmidt^b, 17^	0.73(0.67–0.79)	0.83	0.55	1.84	0.30	0.38
Aekplakorn^18^	0.68(0.62–0.74)	0.54	0.70	1.76	0.67	0.23
Lawati^10^	0.63(0.57–0.69)*	0.85	0.39	1.40	0.39	0.24
Schulze^c, 19^	0.65(0.59–0.71)*	0.73	0.54	1.58	0.51	0.27
León^11^	0.65(0.59–0.71)	0.85	0.39	1.39	0.40	0.24
Wilson^20^	0.63(0.56–0.70)*	0.54	0.66	1.57	0.70	0.20
Balkau^22^	0.65(0.59–0.71)*	0.67	0.57	1.55	0.59	0.24
Bindraban^21^	0.65(0.59–0.71)*	0.48	0.74	1.83	0.71	0.22
Cox^13^	0.67(0.61–0.73)	0.90	0.35	1.39	0.27	0.25

a : The current study did not consider the item “prescribed steroid” that was in the original screening tool;

b : The current study did not consider the item “ethnic” that was in the original screening tool;

c : The current study did not consider the items “ intake of red meat” and “ intake of whole-grain” that were in the original screening tool;

*p<0.05 for comparing the AUC of the specific screening tool with that of ADA.

None of these tools had a positive likelihood ratio greater than or equal to 4 in either men or women. On the other hand, three tools used for males and 10 for females had a negative likelihood ratio less than or equal to 0.6. These useful tools for men were developed by Stern, Mohan, and Leon, and for women, were developed by the ADA, Baan, Griffin, Stern, Schmidt, Lawati, Schulze, Leon, Balkau, and Cox.

## Discussion

In the current study, we evaluated the performance of ADART in predicting pre-diabetes and diabetes based on questionnaires in a prospective cohort of Taiwanese. We found that ADART, which measures self-report variables including age, family history of diabetes, BMI, physical activity, known history of hypertension, gestational diabetes history, and obesity, was a valid tool for predicting the 3-year incidence of pre-diabetes and diabetes, in ethnic Chinese women.

After taking additional demographic factors, lifestyle behaviors, physiological factors and biomarkers into account, the differences in AUCs among these three ROCs were not significant in either men or women. Especially, when biomarkers were added to the model with ADART only, there was no improvement in the prediction of 3-year incidence in both men and women. Because ADART plus biomarkers at baseline did not improve the prediction of the three-year incidence of pre-diabetes and diabetes, compared with ADART only, this may indicate that ADART alone can be applied to the general population for screening pre-diabetes and diabetes, or it may indicate that our study did not have enough power due to the moderate size of the sample. However, effect sizes calculated by the differences in AUC in men and women were 0.04 and 0.03, in sensitivity, 0.06 and 0.02, and in specificity, 0.1 and 0.08, respectively. Given this small magnitude of increase in effect size, there was limited improvement in screening pre-diabetes and diabetes.

An extensive literature review revealed that there are 16 measures for screening and identifying diabetes addition to ADART [8]–[23]. We found that only the tool developed by the Atherosclerosis Risk in Community (ARIC) Study had a higher AUC than that of ADART (0.64 vs. 0.60 in men; 0.73 vs. 0.72 in women), although the difference in the AUC between the two measures was not significant. The AUC for ADART were significantly higher than those for the tools developed by Ramachandran, Aekplakorn, Al-Lawati, Balkau, and Bindraban [9], [18], [10], [21], [22] for men, and by Baan, Lindström, Glumer, Mohan, Romachandran, Al-Lawati, Schulze, Balkau, Bindraban, and Wilson for women [8]–[10], [14], [16], [19], [20], [21], [22], [23]. The predictive ability of ADART indicates that this tool can be used in clinical practice to assist in medical decision-making and to counsel people regarding the likely course of their potential disease. In particular, early lifestyle intervention and counseling should be implemented in order to reduce the risk of disease. We found that the screening program combined with lifestyle behaviors or blood testing performed slightly better in men than in women. Although ADART was developed to be used in white and black populations, this risk assessment tool performed well in this Taiwanese population.

This is the first study to prospectively validate a tool for risk assessment of pre-diabetes and diabetes. Although we used a standardized data collection procedure and evaluated a large number of behavioral factors, we did not perform oral glucose tolerance testing or measure the 2-h glucose concentration. In addition, some of the variables measured with other tools, such as steroid use in Griffin's study and consumption of red meat and whole grain in Schulze's study, were not included when we compared the predictive ability of ADART with that of the other tools.

In conclusion, we found that the use of ADART alone in community screening predicts the 3-year incidence of pre-diabetes and diabetes well in females. Its performance was one of the best among the tools reported in the literature. This was the first testing of this simple screening tool in the Taiwanese population.
